# Female homicides in the state of Maranhão, Brazil, 2000-2019: an ecological study

**DOI:** 10.1590/S2237-96222022000200007

**Published:** 2022-09-19

**Authors:** Sara Ferreira Coelho, Hayla Nunes da Conceição, Andréa Cronemberger Rufino, Alberto Madeiro

**Affiliations:** 1 Universidade Federal do Piauí, Programa de Pós-Graduação em Saúde e Comunidade, Teresina, PI, Brazil; 2 Universidade Estadual do Piauí, Centro de Ciências da Saúde, Teresina, PI, Brazil

**Keywords:** Homicide, Violence Against Women, Mortality, Health Information System, Ecological Studies

## Abstract

**Objective:**

To analyze the profile and temporal trends of female homicides in Maranhão, Brazil, between 2000 and 2019.

**Methods:**

This was an ecological study using data from the Mortality Information System. The profile of the deaths, mortality rate trends (joinpoint method) and correlation with socioeconomic and health indicators (Pearson correlation) were evaluated.

**Results:**

1,915 female homicides were reported, with predominance among those between 20-29 years old (29.9%), single (62.0%), with 4-7 years schooling (29.7%), mixed race (71.3%), homicides at home (31.9%) and by firearms (41.1%). The mortality rate showed a rising trend (APC = +8.21; 95%CI 5.18;10.28). There was negative correlation between homicides and per capita income (p-value = 0.031) and positive correlation with the proportion of families headed by women (p-value = 0.001) and with the rate of male mortality due to assault (p-value = 0.001).

**Conclusion:**

There was an increase in female homicides, related to structural violence in society, poverty and women with greater family authority.

**Study contributions**

**Main results**

Mortality rates showed a rising trend in the state. There was negative correlation between homicides and per capita income and positive correlation with the proportion of families headed by women and the rate of male mortality due to assault.

**Implications for services**

Greater knowledge of the profile of women victims, temporal trend and correlation of deaths with socioeconomic and health indicators can contribute to the development of public policies to protect the most vulnerable women.

**Perspectives**

The continuous improvement of the quality of records and of the Mortality Information System, as well as new epidemiological studies on the theme, will reduce the invisibility and underreporting still typical of feminicides.

## Introduction

Feminicide is the most perverse aspect of gender violence. As a phenomenon present in all societies, violence against women is a public health problem.^1^ Worldwide, approximately 35% of women suffer or have suffered some type of violence, with assault by intimate partners being the most prevalent and common type.^2^ When this violence culminates in death, it is estimated that globally 38% of all feminicides are committed by intimate partners.^1^ The term "feminicide", created to refer to the deaths of women resulting from gender violence, is motivated solely by the fact that the victim is a woman,^3^ which allows us to recognize these deaths as a social and political phenomenon.^4^

The profile of women who suffer violence and/or homicide is represented, in the main, by Black, young, socially and economically vulnerable women, with low education, in unskilled occupations and living in urban regions with little or no security.^5^ ^-^ ^7^ The aggressors, generally, are young, less educated than the women, conjugal partners or acquaintances of the victims, with criminal records and a history of violence.^5^ ^,^ ^6^ Moreover, feminicides are associated with factors such as patriarchal society, economic deprivation, machismo, and/or attempts to assert masculinity over women.^8^ It is noteworthy that the victims often live in regions of greater social inequality, places where there is organized crime, drug trafficking, and places where a high number of homicides of men are registered.^9^

Homicides of women are showing a rising trend worldwide; and the highest rates are found in the Central American countries.^10^ In Brazil, the mortality rate was found to have increased from 5.84 per 100,000 women in 2002 to 6.16 per 100,000 women in 2012, despite the stable trend. Only the Southeast region of the country had a falling rate, of 3.41% per year; while the highest mortality rates were found in states with greater social inequality.^11^ Even with the increase in reported murders in recent years, it is believed that there is still a significant amount of underreporting, especially of homicides occurring outside the family environment, these being situations in which it is difficult to identify the perpetrator, establish the motivation and even register the death.^10^ ^,^ ^12^

Brazilian data on feminicides are still scarce, despite the relevance of the topic. Female homicides in the state of Maranhão, between 2010 and 2015, showed an increase of 52.8% in cases, with an increase of 124.4% in the rate of female mortality due to assault;^13^ however, there is no information specifically about the profile of these women or the evolution/trend of such deaths. 

The objective of this study was to analyze the profile and temporal trend of female homicides in the state of Maranhão, Brazil, in the period from 2000 to 2019.

## Methods

This was an ecological time-series study, having at its unit of analysis the state of Maranhão and its mesoregions. The state has 217 municipalities, distributed over five mesoregions: East, North, Center, West and South. In 2010, 52.2% of the 6,674,789 inhabitants of Maranhão were women, 45.3% of whom had children, 38.5% did not study or work, and 76.4% were of Black race/skin color.^14^

We used secondary data from the Mortality Information System (SIM) and the Brazilian Institute of Geography and Statistics (IBGE) (http://www.datasus.gov.br/mortalidade and http://www.ibge.com.br), accessed in September 2021. We analzyed all cases of female deaths due to assault that occurred between 2000 and 2019, including all age groups. We chose to use female mortality due to assault as a proxy for feminicides in Maranhão, as this was considered to be an alternative to compensate for the high rates of underreporting.^6^ ^,^ ^9^ ^,^ ^15^

The profile of the deaths and the women were assessed according to age group (in years: 0-9; 10-19; 20-29; 30-39; 40-49; 50-59; 60-69; 70 or more), marital status (single; married; separated; other), education (in years of study: none; 1-3; 4-7; 8-11; 12 or more), race/skin color (White; Black; Asian; mixed race; Indigenous), place of occurrence (home; public thoroughfare; health facility), cause of death (one of the ICD-10 codes in the range X85-Y09), and year of death (in five-year periods: 2000-2004; 2005-2009; 2010-2014; 2015-2019).

We calculated the rate of female mortality due to assault in the state, by year, for the entire period from 2000 to 2019, using the classification contained in Chapter XX of the International Statistical Classification of Diseases and Related Health Problems - 10^th^ Revision (ICD-10), according to codes between X85 and Y09. A correction was made to the deaths by means of proportional redistribution of deaths classified as "events of undetermined intent" (Y10-Y34), as per a method used in another study.^16^ To this end, initially we determined the number of deaths due to external causes: accidental falls (W00-X59), intentional self-harm (X60-X84) and legal interventions (Y35). We than calculated the proportion of deaths due to assault (X85-Y09) in relation to total deaths due to external causes. We then multiplied the result by the number of deaths identified as being of "undetermined intent". The result was used as the numerator to calculate the rates of female mortality due to assault. The denominator was the total female population in the age group considered, by year. The result of the division was multiplied by 100,000 women.

The independent variables were divided into socioeconomic indicators and health indicators. The socioeconomic indicators were:

- Gini Index, which expresses the degree of inequality in the distribution of *per capita* household income, ranging from 0 (no inequality) to 1 (maximum inequality);
- Human Development Index (HDI), which expresses the degree of economic development and quality of life of a population, ranging from 0 (no development) to 1 (total development);
- *per capita* income, represented by the ratio between the sum of the *per capita* income of all individuals and the total number of these individuals (in BRL);
- unemployment rate, represented by the ratio between the number of people seeking employment and the number of economically active people in a given period;
- proportion of families headed by women, or percentage of families in which a woman was in charge of the family;
- illiteracy rate, represented by people aged 15 years and older who cannot read and write divided by the total population in the same age group, multiplied by 100; and
- life expectancy at birth, calculated by the average number of years of life expected for a newborn, taking the existing mortality pattern in the resident population, in a given geographical area, in the year considered.

In turn, the health indicators were:

- birth rate, represented by the number of live births, per 1,000 inhabitants, in a given geographic space, in the year under consideration;
- cervical cancer mortality rate, represented by the number of deaths from cervical cancer in residents in a given place, divided by the total resident female population in that place, multiplied by 100,000;
- breast cancer mortality rate, represented by the number of deaths from breast cancer in residents in a given place, divided by the total resident female population in that place, multiplied by 100,000;
- male mortality rate due to assault, represented by the number of male deaths from external causes (assault), divided by the resident male population, multiplied by 100,000; and
- male and female mortality due to ill-defined causes, or the percentage of deaths due to ill-defined causes, by sex, divided by the total number of deaths of resident people, multiplied by 100.

The rate of female mortality due to assault was calculated by year and by mesoregion of the state. Mortality rate trends were assessed using segmented linear regression (joinpoint), with determination of the annual percent change (APC) and 95% confidence intervals (95%CI). We considered that there was an increase in the coefficients when the trend of female mortality due to assault was rising and the minimum value of the 95%CI was greater than 0; and that there was a reduction in female mortality when there was a falling trend, and the maximum value of the 95%CI was less than 0. Stability was defined when, regardless of the trend, the 95%CI included 0. In order to investigate association between the indicators and the mortality rate, we performed bivariate analysis using Pearson’s correlation test, with calculation of the correlation coefficient (r): very weak correlation (0.01-0.19), weak (0.20-0.39), moderate (0.40-0.69), strong (0.70-0.89) and very strong (0.90-0.99). We performed multiple linear regression, with inclusion of variables in the model when the p-value was < 0.20, using the backward stepwise method. Statistical significance was established when the p-value was < 0.05. The analyses were conducted with the aid of the SPSS program (version 20).

As this research used public domain data and with no possibility of the women’s information being indexed, there was no requirement for analysis by a Research Ethics Committee.

## Results

Between 2000 and 2019, 1,915 female deaths due to assault were notified in the state of Maranhão. Table 1 shows that notifications were most frequent among women aged 20 to 29 years (29.9%), single (62.0%), with 4 to 7 years of schooling (29.7%), of mixed race (71.3%). Home was the most frequent place of death (31.9%). The state’s Northern mesoregion had the highest number of notifications in the period covered by the time series (39.9%). The highest proportion of cases (35.0%) was recorded between 2015 and 2019, compared to the other five-year periods. The main means of assault that culminated in the death of women in Maranhão were sharp objects (37.1%) and firearms (X93/X94/X95), the latter accounting for 41.1% of cases.

**Table 1:** Characterization of the notifications of female mortality due to assault, Maranhão, Brazil, 2000-2019

Variables	n	%
**Age group (in years)^a^**		
≤ 9	76	4.0
10-19	264	13.8
20-29	573	29.9
30-39	475	24.8
40-49	248	12.9
50-59	124	6.5
60-69	67	3.5
≥ 70	76	4.0
**Marital status^b^**		
Single	1.187	62.0
Married	290	15.2
Separated	35	1.8
Other	253	13.2
**Schooling (in years of study)^c^**		
None	222	11.6
1-3	305	15.9
4-7	569	29.7
8-11	470	24.5
≥ 12	111	5.8
**Race/skin color^d^**		
White	280	14.6
Black	219	11.4
Asian	3	0.2
Mixed race	1,366	71.3
Indigenous	14	0.7
**Place of occurrence^e^**		
Home	610	31.9
Public thoroughfare	468	24.4
Health facility	520	27.2
Other	297	15.5
**Mesoregion of the state**		
North	761	39.9
West	527	26.5
Center	214	12.8
East	308	15.5
South	105	5.3
**Category (ICD-10)^f^**		
Firearm (X93/X94/X95)	788	41.1
Sharp object (X99)	710	37.1
Blunt object (Y00)	127	6.6
Other unspecified means (Y08/Y09)	99	5.2
Hanging/strangulation/suffocation (X91)	94	4.9
Bodily force (Y04)	30	1.6
Crashing of motor vehicle (Y03)	18	0.9
Other maltreatment (Y07)	12	0.6
Smoke/fire/flames (X97)	9	0.5
Drowning/submersion (X92)	10	0.5
Unspecified chemical or noxious substance (X90)	6	0.3
Sexual assault by bodily force (Y05)	6	0.3
Neglect/abandonment (Y06)	3	0.2
**Period of death**		
2000-2004	247	12.9
2005-2009	353	18.4
2010-2014	645	33.7
2015-2019	670	35.0
Total notifications	1,915	100.0

a) Data unknown: 12 (0.6%); b) Data unknown: 150 (7.8%); c) Data unknown: 238 (12.5%); d) Data unknown: 33 (1.8%); e) Data unknown: 20 (1.0%); f) ICD-10: International Statistical Classification of Diseases and Related Health Problems - Tenth Revision -, namely, specified chemicals and noxious substabces (X89) - 1 (0.066%) -, explosive material (X96) - 01 (0.066%) - and pushing or placing victim before moving object (Y02) - 01 (0.066%); there were no cases of ICD codes X85, X86, X87, X88, X98 or Y01.

All the state’s mesoregions showed an increase in female mortality rates due to assault during the period, with the Southern mesoregion accounting for the largest variation, from 0.78/100,000 women in 2000 to 5.32/100,000 women in 2015 (data not shown). Female mortality rates due to assault in Maranhão showed a rising trend in the period 2000-2019 (APC = +8.21; 95%CI 5.18;10.28); effectively, all the state’s mesoregions showed an upward trend in these rates, with the East (APC = +11.93; 95%CI 9.12;14.84) and the South (APC = +13.10; 95%CI 9.66;17.82) standing out (Table 2).

**Table 2:** Trends in rates of female mortality due to assault, by mesoregion, Maranhão, Brazil, 2000-2019

Maranhão and mesoregions of the state	MR^a^		APC^b^ (95%CI)^c^	p-value^c^	Trend
	2000	2019			
Maranhão	1.53	5.34	8.21 (5.18;10.28)	< 0.001	Rising
North	2.07	5.43	6.92 (4.04;10.32)	< 0.001	Rising
West	1.44	5.48	7.67 (5.15;10.29)	< 0.001	Rising
Center	2.11	4.83	7.32 (3.74;10.78)	0.001	Rising
East	0.50	4.07	11.93 (9.12;14.84)	< 0.001	Rising
South	0.78	5.12	13.10 (9.66;17.82)	< 0.001	Rising

a) MR: Mortality rate; b) APC: Annual percent change; c) 95%CI: 95% confidence interval.

Table 3 shows the socioeconomic and health indicators in 2010, with a description of the mean and minimum and maximum values. In that year, mean *per capita* income in the state was BRL 225.7 and 38.7% of the families residing in Maranhão were headed by women. Also noteworthy was the male mortality rate due to assault: 27.8/100,000.

**Table 3:** Socioeconomic and health indicators of the state of Maranhão, Brazil, 2010

Variables	Mean	Minimum; maximum
**Socioeconomic indicators**		
Gini Index	0.56	0.45;0.72
Human Development Index (HDI)	0.57	0.44;0.76
*Per capita* income (in BRL)	225.70	95.59;770.52
Proportion of families headed by women (%)	38.70	19.22;56.20
Life expectancy at birth (in years)	71.40	67.4;74.7
Unemployment rate (%)	7.40	1.28;22.44
Illiteracy rate (%)	26.39	4.61;38.6
**Health indicators**		
Birth rate (live births/1,000 inhabitantes)	20.85	10.34;29.8
Cervical cancer mortality rate (per 100,000)	16.75	0;32.84
Breast cancer mortality rate (per 100,000)	15.88	0;35.77
Male mortality rate due to assault (per 100,000)	27.82	0.64;31.57
Male mortality rate due to ill-defined causes (%)	8.67	0.78;12.78
Female mortality rate due to ill-defined causes (%)	7.92	0.93;16.48

Table 4 shows the bivariate correlation between the female mortality rate due to assault and socioeconomic and health indicators. Moderate and positive correlation was found between assault due to mortality and the HDI (r = 0.532; p-value = 0.034), the proportion of families headed by women (r = 0.673; p-value < 0.001), unemployment rate (r = 0.477; p-value = 0.001), cervical cancer mortality rate (r = 0.451; p-value = 0.001), breast cancer mortality rate (r = 0.562; p-value = 0.001), male mortality rate due to assault (r = 0.634; p-value < 0.001), and proportion of male deaths from ill-defined causes (r = 0.546; p-value < 0.001). Furthermore, there was a moderate negative correlation between mortality due to assault and *per capita* income (r = -0.658; p-value < 0.001).

**Table 4:** Correlation between the female mortality rate due to assault and socioeconomic and health indicators, Maranhão, Brazil, 2000-2019

Variables	Correlation coefficient (r)	Correlation interpretation	p-value
**Socioeconomic indicators**			
Gini Index	0.045	Very weak	0.624
Human Development Index (HDI)	0.532	Moderate	0.034
*Per capita* income (in BRL)	-0.658	Moderate	< 0.001
Proportion of families headed by women (%)	0.673	Moderate	< 0.001
Life expectancy at birth (in years)	-0.479	Moderate	0.341
Unemployment rate (%)	0.477	Moderate	0.001
Illiteracy rate (%)	0.307	Weak	0.065
**Health indicators**			
Birth rate (live births/1,000 inhabitantes)	-0.253	Weak	0.221
Cervical cancer mortality rate (per 100,000)	0.451	Moderate	0.001
Breast cancer mortality rate (per 100,000)	0.562	Moderate	0.001
Male mortality rate due to assault (per 100,000)	0.634	Moderate	< 0.001
Male mortality rate due to ill-defined causes (%)	0.546	Moderate	< 0.001
Female mortality rate due to ill-defined causes (%)	0.361	Weak	0.356

In the multiple model, after adjustments, three indicators remained associated with female mortality due to assault: the lower the *per capita* income β = -0.553; p-value = 0.031), the higher the mortality coefficient; moreover, the higher the proportion of families headed by women β = 0.637; p-value = 0.001) and the male mortality rate due to assault β = 0.624; p-value = 0.001), the higher the coefficients for mortality due to assault (Table 5). 

**Table 5:** Multivariate analysis between female mortality rate due to assault, socioeconomic and health indicators, Maranhão, Brazil, 2000-2019

Variables	β	95%CI^a^	p-value
**Initial model**			
Human Development Index (HDI)	0.598	0.438;0.635	0.032
*Per capita* income (in BRL)	-0.573	-0.378;-0.796	0.041
Proportion of families headed by women (%)	0.641	0.492;0.845	0.001
Unemployment rate (%)	0.480	-0.897;0.576	0.431
Cervical cancer mortality rate (per 100,000)	0.431	-0.761;0.653	0.538
Breast cancer mortality rate (per 100,000)	0.591	-0.909;0.754	0.281
Male mortality rate due to assault (per 100,000)	0.658	0.451;0.833	0.001
Male mortality rate due to ill-defined causes (%)	0.501	-0.758;0.745	0.221
**Final model**			
Human Development Index	0.573	0.421;0.702	0.423
Renda *per capita* (in BRL)	-0.553	-0.329;-0.843	0.031
Proportion of families headed by women (%)	0.637	0.381;0745	0.001
Male mortality rate due to assault (per 100,000)	0.624	0.398;0.786	0.001

a) 95%CI: 95% confidence interval.

## Discussion

The data produced by this study show that the trend of female mortality due to assault increased in Maranhão in the period from 2000 to 2019. The predominant profile of the victims was that of young adult women, single, with a low level of education, of mixed race, the majority of whom died at home. Moreover, there was negative correlation with *per capita* income; and positive correlation with the proportion of families headed by women and with the rate of male mortality due to assault.

Feminicide is not specified on the Brazilian Death Certificate, making it impossible to identify this type of death in secondary data. In view of this, in this study the concept of female mortality due to assault was used as a proxy for femicides in the state of Maranhão, with correction of deaths classified as "undetermined" in order to calculate the mortality rates.^16^ If, on the one hand, there is a possibility that this indicator overestimates the true numbers of feminicides found, on the other hand, this is an alternative described in the literature as a form of compensating for the underreporting of feminicide.^6^

The rising trend in mortality due to assault was found both for the entire state of Maranhão and also for its mesoregions. It is possible that this fact has occurred not only due to the greater monitoring and visibility that feminicide has gained in society, but also due to the actual increase in female deaths related to gender issues. The data from this study corroborate the increase in the trend of female mortality due to asssault found in the Northeast, Midwest and Southern macro-regions of Brazil between 2002 and 2012.^11^ Moreover, even taking into account regional discrepancies, states with lower HDI tertiles and greater inequality, such as Maranhão and Amazonas, had higher and increasing mortality rates.^11^ In 2014, Maranhão had a higher rate of female mortality due to assault (4.74 per 100,000 women) than both the entire Northeast region (4.05 per 100,000 women) and the entire Southern region (3.82 per 100,000 women) of Brazil.^17^ However, it is necessary to consider that the failure to reduce this type of mortality, even after the implementation of Law No. 13,641, of April 3, 2018, known as Maria da Penha Law, stems from a possible failure in the implementation and enforcement of the law at all stages of the process.^18^

Young and unmarried women were the main victims identified in this research. Other studies confirm that mortality due to assault is responsible for a large portion of deaths of women of reproductive age, with years of potential life lost in the female population, in addition to legal, prison system and public health issues, generating physical and psychological harm for their families.^13^ ^,^ ^17^ ^,^ ^19^ Higher prevalence of femicides among single women has also been found by national studies.^5^ ^,^ ^15^ ^,^ ^19^ In other countries, rates of female mortality due to assault were about eight times higher than those of women who, despite living with their partners, were not formally married.^8^ However, it is known that women, when taking the initiative to seek separation from their partners, husbands and even boyfriends, are more likely to be victims.^1^ ^,^ ^3^ A large part of the population does not consider domestic partnership to be marriage or a legally valid situation and therefore many non-fatal victims of violence have reported that even though they are in domestic partnership relationships, they still consider themselves to be single.^20^

In this research, the most frequent schooling level of murdered women was 4 to 7 years of study. This fact has already been found in previous studies.^6^ ^,^ ^19^ ^,^ ^21^ There is no consensus on the reasons why women having low levels of education is a risk factor for feminicide, but one of the reasons for this result could be that having less formal education results in less empowerment for women and therefore leads them to seek help less frequently in cases of previous violence.^3^ Another characteristic observed was the predominance of women of mixed race as the main victims of homicides, even though in Maranhão, the greater part of the population is Black.^14^ Although there is probably an intersection with other vulnerabilities, among which is the low level of education of this population,^22^ the fact is that, between 2006 and 2013, there was a 2.1% reduction in the number of homicides among White women and a 35% increase among Black women.^23^

The place where assault occurs is a characteristic indicator of feminicides. In this study, the home was found to be the main place of death, followed by healthcare facilities. The high rates of these homicides at home reinforce the indication that these are cases related to violence perpetrated by intimate partners, family members or acquaintances of the victims. Notwithstanding, it can be assumed that these deaths are related to gender issues, characterizing them as feminicides.^3^ ^,^ ^10^ Between 1980 and 2014, there was an equivalent distribution of these deaths between home, hospital and public thoroughfares, in Brazil as a whole and in the Northeast region of the country in particular, with public thoroughfares being the main place where they occurred.^17^ Other studies also point to public thoroughfares as the main place where female homicides occur, followed by homicides in women’s homes, indicating high rates of domestic violence.^22^ ^,^ ^23^ One possibility for the increase in deaths in public thoroughfares would be the fact that many aggressors are ex-husbands and/or ex-partners of the victims and, therefore, know all their routines and assault them on their way from home to work or vice versa.^17^

As found in Maranhão, several studies have shown the predominance of the use of firearms as a means of assaulting women and killing them.^17^ ^,^ ^18^ ^,^ ^22^ In 2015, almost half (48.8%) the homicides of women in Brazil were caused by firearms, despite the increase in homicides by strangulation and/or suffocation, followed by the use of sharp objects.^23^ It is known that the legal possession of a firearm by the perpetrator is one of the risk factors for feminicide,^9^ causing concern about the recent relaxation, promoted by the Brazilian government, of the possession and carrying of firearms. In different places, however, the means employed by the aggressor varies, with a higher prevalence of the use of sharp objects, as observed in Manaus, capital of the state of Amazonas, and in the municipality of Campinas, state of São Paulo.^21^ ^,^ ^24^ In this type of homicide, besides the victim being killed, they are also often disfigured, with a high number of wounds on the face, indicating hate crimes or crimes motivated by banal and futile reasons.^10^ ^,^ ^23^

*Per capita* income showed negative correlation with the coefficient for female mortality due to assault. Maranhão is the Brazilian state with the highest rates of social inequality, with an annual *per capita* household income of BRL 636.00, a 0.639 HDI, the lowest urbanization rate in the country (58.3%), and the highest infant mortality rate (24.7 deaths/1,000 live births).^25^ It is known that societies with higher levels of inequality have higher rates of female mortality due to aggression,^11^ whereby the poorest women are the most affected.^9^There is evidence that places with lower HDIs and greater social inequality have higher rates of female mortality due to assault.^11^ Factors such as unemployment, drug trafficking, family breakdown, and social inequality, especially among youth, exacerbate structural violence and increase women’s vulnerability, with greater impact on gender-based violence.^24^ ^,^ ^26^

Another indicator associated with female mortality due to assault, according to the findings of this study, was the proportion of families headed by women. By subverting traditional gender norms, women challenge the values of a patriarchal society and increase the chance of domestic violence.^4^ On the one hand, the entry of women into the labor market and the changes in social roles have promoted and facilitated female independence. On the other hand, this fact can generate more conflicts with men, since the role of family provider is no longer exclusively male, causing aggressive behavior due to the non-acceptance of this new role for women.^9^ To impose their authority over their female companions, these men often use brute force out of a feeling of inferiority, increasing the risk of feminicide.^27^ ^,^ ^28^

It is well known that deaths due to assault predominate in males, representing about 90% of deaths resulting from violence, with rates 11.5 times higher than those for females.^29^ In addition to magnitude, female homicides are commonly motivated by gender-based issues centered on beliefs of male superiority and exemption from punishment. Despite these differences, places with high rates of male deaths due to assault also have high prevalence of violence against women, which can result in female homicides,^29^ as found in our study. Younger men, those who live in areas of social vulnerability, who abuse alcohol and illicit drugs, as well as being perpetrators of gender-based violence, are more likely to die due to assault.^17^ In Brazil, between 2010 and 2014, the rate of male mortality due to assault was 133.3/100,000 inhabitants and in Maranhão, during the same period, the rate was higher than the national rate (158.2/100,000 inhabitants). The large number of unemployed young people, who did not attend or do not attend school and who come from broken homes, are factors that contribute to the increase in violence^30^ and gender crimes.^5^ ^,^ ^6^ ^,^ ^11^ ^,^ ^15^ ^,^ ^19^

This study has limitations that should be highlighted. Firstly, given that it is a study based on secondary data, only the variables available on the death certificate were used, hindering a more comprehensive understanding of this multidimensional phenomenon. Moreover, the incompleteness of some variables favors data invisibility and hinders the analysis of the real pattern of homicides. Secondly, it was not possible to know what motivated the female homicides, since no data regarding the aggressor is held on the Mortality Information System, or whether what led to the deaths of these women were gender issues. When correcting for deaths with undetermined causes, there is a chance of overestimating feminicides. Nevertheless, this is an alternative used to compensate for the frequent underreporting of female mortality due to assault. Thirdly, the possibility of ecological fallacy, typical of the design of this study, must be considered. Thus, it may be inappropriate to generalize the results obtained from the population of the state as a whole to the individuals within it.

Even with these limitations, this study is pioneer in using information on the death of women due to assault in Maranhão. The data obtained may allow the development of public policies for the protection of women and the improvement of strategies to refine the quality of the records. As a serious and shameful phenomenon for any society, a more reliable view of feminicides is a necessary strategy for their eradication. Despite progress with legislation to protect women, an increase in cases of female homicides can be seen. When one takes into account the invisibility and underreporting typical of this phenomenon, the data from this study gains even more relevance. These results reinforce both the magnitude and the omissions that mark the trajectory of the murders of women, indicating the need for new epidemiological studies for a better understanding and continuous surveillance of feminicides. 
